# Disturbance-Disturbance uncertainty relation: The statistical distinguishability of quantum states determines disturbance

**DOI:** 10.1038/s41598-018-22336-3

**Published:** 2018-03-05

**Authors:** E. Benítez Rodríguez, L. M. Arévalo Aguilar

**Affiliations:** Facultad de Ciencias Físico Matemáticas, Benemérita Universidad Autónoma de Puebla, 18 Sur y Avenida San Claudio, Col. San Manuel, C.P: 72520 Puebla, Pue. Mexico

## Abstract

The Heisenberg uncertainty principle, which underlies many quantum key features, is under close scrutiny regarding its applicability to new scenarios. Using both the Bell-Kochen-Specker theorem establishing that observables do not have predetermined values before measurements and the measurement postulate of quantum mechanics, we propose that in order to describe the disturbance produced by the measurement process, it is convenient to define disturbance by the changes produced on quantum states. Hence, we propose to quantify disturbance in terms of the square root of the Jensen-Shannon entropy distance between the probability distributions before and after the measurement process. Additionally, disturbance and statistical distinguishability of states are fundamental concepts of quantum mechanics that have thus far been unrelated; however, we show that they are intermingled thereupon we enquire into whether the statistical distinguishability of states, caused by statistical fluctuations in the measurement outcomes, is responsible for the disturbance’s magnitude.

## Introduction

The Heisenberg uncertainty principle (HUP) is related in a complex form to other fundamental quantum phenomena and difficult concepts of quantum mechanics. For example, it is closely related to quantum measurement and state’s preparation^1^. Also, it is related to the stability of matter^2^, complementarity^3,4^, entanglement^5–10^, and, recently, it was shown that the strength of the uncertainty principle (together with the strength of steering) underlies quantum non-locality^11^. Currently, as far as we are aware, there are at least three generic types of uncertainty principles^1^, where every single one has its own uncertainty relation (M.J.W. Hall acknowledges four generic types of uncertainty principles^3^).

According to the convention adopted in this paper, we should stress that we use the term *uncertainty relation* to mean the mathematical expression of the uncertainty principle, as done for example by Uffink and Hilgevoord^12^. This convention takes into account the distinction between *preparation* and *measurement*^13–15^. For example, using this convention it could be interpreted that Busch *et al*.^1^ list three types of HUP: A) *It is impossible to prepare states which possess two non-commuting observables simultaneously arbitrarily well localized*, B) *It is impossible to measure simultaneously two non-commuting observables*, and C) *It is impossible to measure one observable without disturbing a non-commuting observable*. Thus, regarding this convention, the HUP given in A) refers to preparation of states, B) refers to simultaneous measurement and C) to the disturbance caused by the measurement process. In this sense, A) and B) are bonded up to the different notions of *preparation* and *measurement* respectively. Each one of these types have its own uncertainty relation, for example: (a) $\delta \hat{A}\delta \hat{B}\ge \hslash |\langle \psi |[\hat{A},\hat{B}]|\psi \rangle |\mathrm{/2}$^16^, (b) $\varepsilon (\hat{A})\eta (\hat{B})\ge \hslash |\langle \psi |[\hat{A},\hat{B}]|\psi \rangle |\mathrm{/2}$^16^, (c) $\varepsilon (\hat{A})\varepsilon (\hat{B})\ge \hslash |\langle \psi |[\hat{A},\hat{B}]|\psi \rangle |\mathrm{/2}$^16^, respectively. Additionally, there exist the entropic uncertainty relation (EUR) whose initial purpose was to overcome the state’s dependence in the uncertainty relations^17–19^. For instance, the Deutsch’s EUR^19^ is linked with preparation of states and it does not refer to the disturbance process; on the other hand, the information-disturbance tradeoff^20^ refers to the disturbance process and the extraction of information.

It is worth mentioning that we do not claim it as the best convention, however it serves to differentiate between the preparation and the measurement processes. It differs from the D’Ariano adopted convention^21^, where “uncertainty relations” are associated with measurements on an ensemble, whereas the “uncertainty principle” is associated with a sequence of measurements on the same system. In another convention the term “uncertainty principle” is often referred to the information gained and the state change induced by the measurement process, whereas the term “uncertainty relations” relates the statistics of the measured observable to the statistics of a non-commuting one.

One of the above HUP formulation refers to the disturbance’s process, which is of paramount importance in the applications of quantum information; hence, many approaches have been developed to define disturbance. As disturbance is one of the major themes in this paper; then, it is convenient to review some previous defined *disturbance* notions. As a result of the vast and rather extensive bibliography regarding this topic, in this paper we make an arbitrary short selection of some representative works. In the *noise-disturbance* relation^16^ the effort was focused on precisely define both noise and disturbance and to differentiate them from the standard deviation. In this approach, Ozawa^16^ initially defined disturbance in terms of what he called the disturbance operator, i.e. $\hat{D}(\hat{B})={\hat{B}}^{out}-{\hat{B}}^{in}$ see reference^16^ for details. This was a state-dependent definition that some years later was redefined by Buscemi *et al*.^22^ in terms of the conditional entropy to get an state independent definition focused on the loss of correlation introduced by the change in the system’s dynamical variables, i.e. disturbance is defined with respect to two system observables. On the other hand, Busch *et al*.^23^ gave a proof of an uncertainty relation for position and momentum based on what they called calibrated error, in this case the disturbance is defined as the root mean square deviation from a sharp value of the observable.

Also, disturbance was associated with the possibility of probabilistically undoing the measurement that causes it^21^, and a tradeoff intimately linked to the impossibility of determining the state of a single system was proposed. This lead to define the gained information as the difference between the Shanon entropy before and after the measurement and disturbance summed up the amount of how the input state is unitarily uncorrelated with the output state; in this sense, disturbance sizes the inability of approximately reverse a measurement and it must only be a function of the probabilities of reversing it.

Buscemi *et al*.^24^ acknowledge the fundamental importance for quantum mechanics and quantum information in developing a universal relation between information extraction and disturbance; to accomplish this task, they proposed genuine quantum quantities to define both quantum information gain and quantum disturbance. Thus, as coherent information (CI) is related to the possibility of constructing a recovery operation, in order to define disturbance they generalize CI (previously used by Maccone to define disturbance^25^) which is related to undoing the state change.

Additionally, the information-disturbance tradeoff has been extended to continuous variables. In this respect, Paris^26^ analyses the information-disturbance tradeoff for continuos variables presenting a scheme to quantify the information gained and the induced disturbance by coupling the system to a single probe system; here, disturbance is defined in terms of the transmission fidelity^26^.

Recent studies have approached the uncertainty relations from quantum estimation theory introducing a noise-noise uncertainty relation^27^, which is of great relevance for our work, see also^28^. Following this approach, in a recent work, noise was defined in terms of the classical Fisher information and disturbance in terms of the quantum Fisher information^29^; also, in this work^29^, it was presented an information-disturbance relation based on divergences mentioning the work of Barchielli and Lupiere^30^, where initially was used the relative entropies both classical and quantum^30^ and extending them towards an arbitrary divergence. However, as it is well know, the relative entropy is not symmetric. This latter approach is quite related to the approach carried out in our work.

In the information-disturbance setting, disturbance’s definition is classified in at least two ways^31^:(i) how close the initial and final states are in terms of the average output fidelity, and (ii) how reversible (or coherent) is the transformation causing the state change. More recently, these approaches have been classified into two different types^29^: (a) an information-theoretic approach, and (b) an estimation-theoretic approach. However, in a more general setting, all the previous works can be classified attending the two distinct relevant properties focused on the observables, as follows: (I) *noise-disturbance* uncertainty relations, e.g. in^16,22^, (II) *information-disturbance* uncertainty relations, e.g. in^21,24,26,29^, and (III) *noise-noise* uncertainty relation, e.g. in^27,28^. Here, in this work we will pursuit the idea of a new relation: (IV) *disturbance-disturbance* uncertainty relation.

Hence, one of the core concepts of uncertainty relations is *disturbance*^16,20–26,32–49^, that is, the strength of the measurement process to cause a perturbation. This is a key concept, needed in order to understand quantum cryptography^39,10^, i.e. to prevent information from being eavesdropped and to analyse security issues^38^, quantum teleportation^34,39^, quantum cloning^39^, entanglement^38,33^, and many facets of the HUP.

On the other hand, another fundamental and truly relevant concept of quantum theory is the *statistical distinguishability of states* in the way that was conceived by Wootters^50^. Then, disturbance and states’ statistical distinguishability^50^ are two core concepts of quantum mechanics that, to the best of our knowledge, have so far been unrelated.

Then, in this paper, we propose that *disturbance* could be characterized by the concept of statistical distinguishability of quantum states. To show this, we use the following two facts: (1) the complete set of the postulates of quantum mechanics (especially the measurement postulate) and (2) the underlying principle^32,51,52^, that claims that observables do not possess pre-existing values before measurements (the effort of Einstein to circumvent the uncertainty principle and nonlocality^5^, the Bell-Kochen-Specker theorem to test it^32,51–54^, and the experimental works of Aspect *et al*.^55^ lay the theoretical and experimental foundation for this principle) to capture the essence of the *disturbance* produced on quantum states while measuring an observable. This leads us to pursuit an uncertainty relation that capture the relation given in (IV) using the following idea:

*It is impossible to measure an observable without disturbing simultaneously its probability distribution and the probability distribution of a non-conmuting observable*.

The previous reasons lead us to propose a definition of *disturbance* based on the distance between two probability distributions (which we call statistical distributions also), the distance will be measured by the square root of the Jensen-Shannon entropy^56^. This definition allows us to uncover a Disturbance-Disturbance uncertainty relation. Also, this definition could be used in the noise-disturbance uncertainty relation^16,22,23,32,40,41,57^, by adapting our definition of disturbance to define the noise process. On the other hand, our approach also could be generalised to the form of root-mean-square deviation^57^ uncertainty relations. Additionally, our definition could be useful with regard to the information-gain-disturbance uncertainty relation^20,21,24–26,35,37,45,47,48^. Our approach also could be generalised to include more that two observables^58–60^, likewise as the Jensen-Shanon entropy was generalised to continuos variables^56^, then it also could be generalised to the case of continuos variables^26^. However, all this requieres further studies and calculations.

The postulates of quantum mechanics are indispensable to clearly understand our treatment, since they establish one of the two facts on which our approach is based. For a modern statement of the postulates of quantum mechanics see the papers by Paris^61^ and Bergou^62^. Here we are going to focus mainly on the measurement postulate only^63^:

*Measurement postulate (MP):* In the measurement process the wave function suffers an abrupt change towards the eigenfunction associated with the determined eigenvalue. That is, if the eigenvalue *a*_*k*_ is obtained when measuring the observable $\hat{A}$, then the wave function collapses as $|{{\rm{\psi }}}_{i}\rangle \to |{a}_{k}\rangle$; where $|{{\rm{\psi }}}_{i}\rangle$ is the state immediately before the measurement^63^.

Additionally, it is worthy of mention the following corollary which is implied by the quantum measurement postulate:

Corollary 1: The *Measurement postulate* allows measurements without collapsing the wave function, since if $|{{\rm{\psi }}}_{i}\rangle =|{a}_{k}\rangle$, then there is not any collapse when measuring $\hat{A}$. Instead, immediately after the measurement, the wave function remains in the same initial state $|{{\rm{\psi }}}_{i}\rangle =|{a}_{k}\rangle$^63^. Then, as this imply that the statistical distribution does not change, we exclude this case in this work.

It is important to mention that to obtain the usual textbook uncertainty relation, it is necessary to use just a few postulates of quantum mechanics (in particular excluding the MP) and the Schwarz inequality; however, it is related to preparation of states but not to the measurement process^64,65^, because the MP is not used to deduce it. *In fact, the deduction of many uncertainty relations does not use the MP*. Also, the entropic uncertainty relations^17–19^, in terms of the Shannon entropy could be obtained without using the MP. Consequently, many of the entropic uncertainty relations are also related to preparation of states only, but not to measurement. As our proposal to define disturbance is a relation given in terms of entropy, it will be valid to use it in the description of the tradeoff between noise and disturbance, taking the noise as the statistical distance between the expected distribution and the experimental distribution^57^.

To illustrate our approach better we use the following example: suppose that you have an initial wave function ${\psi }_{i}(x)={c}_{1}{e}^{-{x}^{2}/4{\sigma }^{2}}$, then you measure the observable $\hat{x}$ and you obtain the eigenvalue *x*_*s*_. Due to the MP, after the measurement the wave function collapses towards the eigenfunction associated with the obtained eigenvalue, i.e. *ψ*_*i*_(*x*) → *ψ*_*f*_(*x*_*s*_). Consequently, the complementary observable $\hat{p}$ has evolved from having no predetermined value in the state *ψ*_*i*_(*x*) to “get”, also, no predetermined value at the final state *ψ*_*f*_(*x*_*s*_). Then, the following questions arise: What was disturbed? Was the disturbance of $\hat{p}$ from having no predetermined value in *ψ*_*i*_(*x*) to getting no predetermined value in *ψ*_*f*_(*x*_*s*_)? How we can measure the disturbance between ${\hat{p}}_{i}$ and ${\hat{p}}_{f}$ when both of them do not possess a predetermined value? What is ${\hat{p}}_{i}$? and What is ${\hat{p}}_{f}$? Those kind of questions suggest that it could be useful to consider that *disturbance* is on the wave function, as the MP implies. Accordingly, it would be interesting to pursuit this approach and associate disturbance with a metric distance between *ψ*_*i*_(*x*) and *ψ*_*f*_(*x*_*s*_). This goal is what we carry out in this paper. Then, in the subsequently sections, we suppose that disturbance occurs on the system’s state, this supposition is based on the following two reasons: i) observables do not have a pre-existing value, ii) The *MP* establishes a change on the system’s state.

Additionally, notice that although there are three equivalent quantum mechanical pictures, i.e. the Schrödinger, Heisenberg, and Interaction pictures, usually the postulates are stated in the Schrödinger picture only. To the best of our knowledge, there is not a statement of the MP in the Heisenberg or Interaction pictures. That is to say, we do not know the equivalent of the MP in the Heisenberg picture.

*Our proposal*. - The thought experiment we are considering is the following: there is a quantum system prepared in a quantum state, its properties are represented by self-adjoint operators^66^. Then, we carry out a single projective measurement of one property, e.g. $\hat{A}$. Hence, we disturb the state of the system, and due to this single measurement the state of the system collapses towards an eigenstate of the observable $\hat{A}$, as is prescribed by the *MP*. Therefore, because this disturbance is on the state of the system, there is a new probability distribution associated with $\hat{B}$.

## Results

In order to capture the disturbance caused by the measurement process, we will proceed to compare the distance between the statistical distribution of the observable $\hat{A}$ before the single measurement and the statistical distribution of the same observable after that single measurement. Also, we compare the distance between the probability distributions of observable $\hat{B}$ before and after the measurement of observable $\hat{A}$. Then, *we define the disturbance caused by the process of measurement as the distance between the probability distribution before and the probability distribution after the measurement process*. Consequently, one of the main goals of this paper is to show how the sum of these distances has an irreducible lower bound. The idea of quantifying disturbance as the distance between probability distributions is not new, it appears clearly stated by Werner^67^ *as the distance between probability measures as the largest difference of expectation values*, also it was already stated by Bush^68^, see also^23^.

Because it obeys the triangle inequality, a good measure of the distance between two discrete probability distributions is the square root of the symmetric Jensen-Shannon entropy^56^:

1 $${D}_{PQ}=\sqrt{\sum _{j=1}^{N}({p}_{j}\,\mathrm{ln}\,\frac{2{p}_{j}}{{p}_{j}+{q}_{j}}+{q}_{j}\,\mathrm{ln}\,\frac{2{q}_{j}}{{q}_{j}+{p}_{j}})},$$

where *p*_*j*_ and *q*_*j*_ are two probability distributions. In this case, we associated *p*_*j*_ with the initial probability distribution, i.e. before the measurement, and *q*_*j*_ with the final probability distribution, i.e. after the measurement.

Then, we get the Jensen-Shannon entropy in terms of the eigenstates of the observables. That is to say, for the observable $\hat{B}$ we have the association ${p}_{j}={|\langle {b}_{j}|\psi \rangle |}^{2}$ and ${q}_{j}={|\langle {b}_{j}|{a}_{s}\rangle |}^{2}$, where $|\psi \rangle$ is the initial state immediately before the measurement and $|{a}_{s}\rangle$ is the state after the measurement of observable $\hat{A}$ associated with the eigenvalue *a*_*s*_ obtained in the measurement process. This association refers to the possibility that the resultant metric space can be embedded in a real Hilbert space^69–71^.

In order to compare the probability distribution of observable $\hat{B}$ before the measurement versus the probability distribution of the same observable after that measurement (of $\hat{A}$) we use the Jensen-Shanon entropy. Consequently, we take ${D}_{\hat{B}}$ as the disturbance in the statistical distribution of $\hat{B}$ because of the measurement of $\hat{A}$, and we find it as:

2 $${D}_{\hat{B}}=\sqrt{\sum _{j=1}^{N}(|\langle {b}_{j}|{a}_{s}\rangle {|}^{2}\,\mathrm{ln}\,\frac{2|\langle {b}_{j}|{a}_{s}\rangle {|}^{2}}{|\langle {b}_{j}|{a}_{s}\rangle {|}^{2}+|\langle {b}_{j}|\psi \rangle {|}^{2}}+|\langle {b}_{j}|\psi \rangle {|}^{2}\,\mathrm{ln}\,\frac{2|\langle {b}_{j}|\psi \rangle {|}^{2}}{|\langle {b}_{j}|\psi \rangle {|}^{2}+|\langle {b}_{j}|{a}_{s}\rangle {|}^{2}})},$$

where |〈*b*_*j*_|*ψ*〉|^2^ is the probability of $\hat{B}$ before the measurement, and |〈*b*_*j*_|*a*_*s*_〉|^2^ is the probability of $\hat{B}$ given that the state after the measurement is an eigenvector of $\hat{A}$, i.e. $|{a}_{s}\rangle$. A similar approach is used to define the disturbance of $\hat{A}$, i.e. ${D}_{\hat{A}}$, as:

3 $${D}_{\hat{A}}=\sqrt{\sum _{j=1}^{N}(|\langle {a}_{j}|{a}_{s}\rangle {|}^{2}\,\mathrm{ln}\,\frac{2|\langle {a}_{j}|{a}_{s}\rangle {|}^{2}}{|\langle {a}_{j}|{a}_{s}\rangle {|}^{2}+|\langle {a}_{j}|\psi \rangle {|}^{2}}+|\langle {a}_{j}|\psi \rangle {|}^{2}\,\mathrm{ln}\,\frac{2|\langle {a}_{j}|\psi \rangle {|}^{2}}{|\langle {a}_{j}|\psi \rangle {|}^{2}+|\langle {a}_{j}|{a}_{s}\rangle {|}^{2}})},$$

notice that 〈*a*_*j*_|*a*_*s*_〉 = *δ*_*js*_. It is important to emphasise that our framework only applies to projective measurements.

In order to find out if there is a minimum, different from zero, of the sum ${D}_{\hat{A}}+{D}_{\hat{B}}$, we take into account how the distance between the probability distributions, represented by *P* and *Q*, behaves when one of the probability distributions tends to the other, i.e. we need to consider the following limit:

4 $$\mathop{\mathrm{lim}}\limits_{P\to Q}{D}_{PQ}^{2},$$

which, expanded term by term, tends to the second order of *p*_*j*_:^56^

5 $$\frac{1}{4}{\chi }^{2}=\frac{1}{4}\sum _{j}\frac{{({p}_{j}-{q}_{j})}^{2}}{{q}_{j}}.$$

This is the *χ*^2^-distance between *p*_*j*_ and *q*_*j*_. However, that distance is not symmetric and we can express it in terms of the probability distribution of $\hat{B}$. Due to this asymmetry, the two *χ*^2^ distances that we can get are the following,

6 $$\frac{1}{4}{\chi }^{2}(|\langle {b}_{j}|{a}_{s}\rangle {|}^{2},|\langle {b}_{j}|\psi \rangle {|}^{2})=\frac{1}{4}\sum _{j}|\langle {b}_{j}|{a}_{s}\rangle {|}^{2}{(1-\frac{|\langle {b}_{j}|\psi \rangle {|}^{2}}{|\langle {b}_{j}|{a}_{s}\rangle {|}^{2}})}^{2},$$

7 $$\frac{1}{4}{\chi }^{2}(|\langle {b}_{j}|\psi \rangle {|}^{2},|\langle {b}_{j}|{a}_{s}\rangle {|}^{2})=\frac{1}{4}\sum _{j}|\langle {b}_{j}|\psi \rangle {|}^{2}{(1-\frac{|\langle {b}_{j}|{a}_{s}\rangle {|}^{2}}{|\langle {b}_{j}|\psi \rangle {|}^{2}})}^{2}.$$

At this point, we are going to find out the value of the *χ*^2^-distance given by equations (6) and (7) based on the statistical distinguishability criterion defined by Wooters^50^, and consequently proving that it is different from zero. We recall that we are considering the case where the initial state is different from an eigenstate of $\hat{A}$, i.e. $|\psi \rangle \ne |{a}_{j}\rangle ,\forall {j}$.

To find out the value of the *χ*^2^-distance in terms of the statistical distinguishability criterion we make the following consideration: *the disturbance should be minimal if the initial state* $|{\psi }_{N}\rangle$ *immediately before the measurement is only slightly different from the final state after the measurement, i.e. if the initial state is the nearest distinguishable neighbour of* $|{a}_{s}\rangle$ ^50,72^. Physically, this means that the measurement process projects the state $|{\psi }_{N}\rangle =|{a}_{s}\rangle +|\delta {a}_{s}\rangle$ to the nearest neighbour distinguishable state $|{a}_{s}\rangle$, i.e. they are the nearest neighbour statistical distinguishable states^50,72^, expressed in an orthonormal basis: $|{a}_{s}\rangle =\sum \sqrt{{p}_{j}}{e}^{i{\varphi }_{j}}|j\rangle$ and $|{a}_{s}\rangle +|\delta {a}_{s}\rangle =\sum \sqrt{{p}_{j}+\delta {p}_{j}}{e}^{i({\varphi }_{j}+\delta {\varphi }_{j})}|j\rangle$^72^. In other words, the disturbance is minimum when the probability distributions (before and after the measurement) of the possible outcomes are the closest statistically distinguishable distributions.

Wootters defines the statistical distance to distinguish between preparation of quantum states^50^. Here, we are using his distinguishability criterion to define the disturbance as the number of statistical distinguishable states between the initial state before measurement and the collapsed state after measurement (we take the minimum disturbance as the distance between the nearest neighbour statistical distinguishable states); that is, by taking disturbance as the distance between distinguishable probability distributions. Wootters proves that the statistical distance for preparation of states, which is determined by statistical fluctuations, is equivalent to the distance between pure states, i.e. the angle between rays. This distinguishability criterion determines that two probability distributions are distinguishable in *n* trials if the following condition is fulfilled:

8 $$\frac{\sqrt{n}}{2}{\{\sum _{i}\frac{{(\delta {p}_{i})}^{2}}{{p}_{i}}\}}^{1/2} > 1,$$

this, in turn, establishes a distance given by^69^:

9 $$ds=\frac{1}{2}\sqrt{\sum _{i}\frac{{(\delta {p}_{i})}^{2}}{{p}_{i}}},$$

where *δp*_*i*_ refers to the difference between the two probability distributions being considered. Equation (8) comes from the Local Limit Theorem^50,73^, whe*n n* → ∞. Additionally, it was demonstrated by Majtey *et al*.^69^ that the distinguishability criterion of Wootters and the one generated by the Jensen-Shanon entropy are almost identical (up to fourth order) for two close enough distributions. Moreover, Majtey *et al*.^69^ have shown that the distance of Wootters is an upper bound for the Jensen-Shanon entropy. Later, Briët *et al*.^70^ completed the proof that the Jensen-Shanon entropy fulfills the requirements of a metric distance and they called it the *transmission metric*, because it is associated with the rate of transmission for a discrete memoryless channel.

Notice that equation (9) (which sets the Wootters distinguishability criterion) is equal to the square root of equation (5), the latter comes from taking the limit *p*_*j*_ → *q*_*j*_ on the Jensen-Shannon entropy, i.e. between the nearest probability distributions. This fact allows us to measure the amount of disturbance by using the distance generated by the Wootters statistical distinguishability of quantum states, i.e. by counting the number of distinguishable states between the states before and after the measurement process. This distinguishability should be defined by the statistical result of measurements that resolve the nearest neighbour states. In fact, Majtey *et al*. established a distinguishability criterion after *n* trials based on the Jensen-Shannon entropy^69^, i.e. ${D}_{\hat{B}}\ge 1/\sqrt{2n}$; however, this criterion is equal to the Wootters criterion given in equation (8) for sufficiently closed enough probability distributions, see section 3 of reference^69^ for details.

Therefore, to find out the value of *χ*^2^ based on the statistical distinguishability criterion of Wootters, we take the state immediately before the measurement as $|{\psi }_{N}\rangle =|{a}_{s}\rangle +|\delta {a}_{s}\rangle$, and normalized. So, we can write equations (6) and (7), respectively, as

10 $$\frac{1}{4}{\chi }^{2}(|\langle {b}_{j}|{a}_{s}\rangle {|}^{2},|\langle {b}_{j}|{\psi }_{N}\rangle {|}^{2})\ge \frac{1}{4}\sum _{j}|{c}_{j}^{s}{|}^{2}{(1-\frac{|{c}_{j}^{s}{|}^{2}+(\delta |{c}_{j}^{s}{|}^{2})}{|{c}_{j}^{s}{|}^{2}})}^{2}={\chi }_{\hat{B},{\min }}^{s(1)},$$

11 $$\frac{1}{4}{\chi }^{2}(|\langle {b}_{j}|{\psi }_{N}\rangle {|}^{2},|\langle {b}_{j}|{a}_{s}\rangle {|}^{2})\ge \frac{1}{4}\sum _{j}(|{c}_{j}^{s}{|}^{2}+\delta |{c}_{j}^{s}{|}^{2}){(1-\frac{|{c}_{j}^{s}{|}^{2}}{|{c}_{j}^{s}{|}^{2}+(\delta |{c}_{j}^{s}{|}^{2})})}^{2}={\chi }_{\hat{B},{\min }}^{s(2)},$$

where ${c}_{j}^{s}=\langle {b}_{j}|{a}_{s}\rangle$, $\delta {c}_{j}^{s}=\langle {b}_{j}|\delta {a}_{s}\rangle$ and $\delta |{c}_{j}^{s}{|}^{2}=|\langle {b}_{j}|\delta {a}_{s}\rangle {|}^{2}$. Notice that

12 $$|\delta {a}_{s}\rangle =|{\psi }_{N}\rangle -|{a}_{s}\rangle ,$$

i.e. the norm ||(|*δa*_*s*_〉)||^2^ is the distance between the states $|{\psi }_{N}\rangle$ and $|{a}_{s}\rangle$, when they are the nearest neighbours, and in some sense this distance represents a unit (statistical distinguishable) to measure distances between states.

Thus, we get the following relations for the distinguishability distance^50,72^, between two different probability distributions, caused by the disturbance of the measurement process:

13 $${D}_{\hat{B}}\ge \frac{1}{2}\sqrt{{\chi }^{2}(|\langle {b}_{j}|{a}_{s}\rangle {|}^{2},|\langle {b}_{j}|\psi \rangle {|}^{2})}\ge \sqrt{{\chi }_{\hat{B},{\min }}^{s(1)}},$$

14 $${D}_{\hat{B}}\ge \frac{1}{2}\sqrt{{\chi }^{2}(|\langle {b}_{j}|\psi \rangle {|}^{2},|\langle \langle {b}_{j}|{a}_{s}\rangle {|}^{2})}\ge \sqrt{{\chi }_{\hat{B},{\min }}^{s(2)}},$$

where ${D}_{\hat{B}}$ is expressed in equation (2), equations (13) and (14) exist because the distance *χ*^2^ is not symmetric. For practical purposes we can take the minimum of $\sqrt{{\chi }_{\hat{B},{\min }}^{s(1)}}$ and $\sqrt{{\chi }_{\hat{B},min}^{s\mathrm{(2)}}}$ as ${\rm{\min }}\{\sqrt{{\chi }_{\hat{B},min}^{s\mathrm{(1)}}},\sqrt{{\chi }_{\hat{B},min}^{s\mathrm{(2)}}}\}$, and we take into consideration only one of these relations. The minimum refers to the quantification of a unit of distance, employing the nearest distinguishable neighbour probability distributions.

Notice that experimentally the minimum must be chosen in such a way that equations (13) and (14) arise from a calibration process, of the measurement apparatus, to find out the nearest statistical distinguishable states of the measured observable $\hat{A}$.

Hence, we can write down our first result as the following equation, which we named the *Jensen-Shannon entropy relation for disturbance*

15 $${D}_{\hat{B}}\ge \,{\rm{\min }}\{\sqrt{{\chi }_{\hat{B},min}^{s\mathrm{(1)}}},\sqrt{{\chi }_{\hat{B},min}^{s\mathrm{(2)}}}\}\mathrm{.}$$

As a final step to complete our study, we calculate the distance between the probability distributions of observable $\hat{A}$ before and after the measurement process. We recall that in our thought experiment we measure the observable $\hat{A}$, then the state of the system collapses towards an eigenstate of the same observable. Then, the probability of finding the system in an eigenstate of $\hat{A}$ before the measurement is |〈*a*_*j*_|*ψ*〉|^2^. In addition, after the measurement we can say with absolute certainty that the system is in an eigenstate of $\hat{A}$, say |*a*_*s*_〉; where the probability of finding the system in |*a*_*s*_〉 is 1.

Carrying out the same process used to obtain ${D}_{\hat{B}}$, we know from equations (4) and (5) that the Jensen-Shannon entropy for ${D}_{\hat{A}}$ tends to *χ*^2^ when one distribution tends to the other, so we have:

16 $${D}_{\hat{A}}\ge \frac{1}{2}\sqrt{{\chi }^{2}(|\langle {a}_{j}|\psi \rangle {|}^{2},{\delta }_{j,s})}\ge \sqrt{{\chi }_{\hat{A},min}^{s\mathrm{(1)}}},$$

17 $${D}_{\hat{A}}\ge \frac{1}{2}\sqrt{{\chi }^{2}({\delta }_{j,s},|\langle {a}_{j}|\psi \rangle {|}^{2})}\ge \sqrt{{\chi }_{\hat{A},min}^{s(2)}},$$

where

18 $$\frac{1}{4}{\chi }^{2}(|\langle {a}_{j}|\psi \rangle {|}^{2},{\delta }_{j,s})\ge \frac{1}{4}\sum _{j}({\delta }_{j,s}+(\delta |{d}_{j}^{s}{|}^{2})){(1-\frac{{\delta }_{j,s}}{{\delta }_{j,s}+(\delta |{d}_{j}^{s}{|}^{2})})}^{2}={\chi }_{\hat{A},min}^{s\mathrm{(1)}},$$

19 $$\frac{1}{4}{\chi }^{2}({\delta }_{j,s},|\langle {a}_{j}|\psi \rangle {|}^{2})\ge \frac{1}{4}\sum _{j}{\delta }_{j,s}{(1-\frac{{\delta }_{j,s}+(\delta |{d}_{j}^{s}{|}^{2})}{{\delta }_{j,s}})}^{2}={\chi }_{\hat{A},min}^{s\mathrm{(2)}},$$

where $\delta {d}_{j}^{s}=\langle {a}_{j}|\delta {a}_{s}\rangle$, and with similar definitions as those given after equations (10) and (11). These last two equations can be reduced, so that

20 $${\chi }_{\hat{A},min}^{s\mathrm{(1)}}=\frac{1}{4}(1+(\delta |{d}_{s}{|}^{2})){(1-\frac{1}{1+(\delta |{d}_{s}{|}^{2})})}^{2}+\frac{1}{4}\sum _{k\ne s}(\delta |{d}_{k}{|}^{2}),$$

21 $${\chi }_{\hat{A},min}^{s\mathrm{(2)}}=\frac{1}{4}{((\delta |{d}_{s}{|}^{2}))}^{2}\mathrm{.}$$

Finally, we obtain the *Entropic Uncertainty Relation of Disturbance-Disturbance* as the sum of the disturbance of observables $\hat{A}$ and $\hat{B}$,

22 $${D}_{\hat{A}}+{D}_{\hat{B}}\ge \,{\rm{\min }}\{\sqrt{{\chi }_{\hat{A},min}^{s\mathrm{(1)}}},\sqrt{{\chi }_{\hat{A},min}^{s\mathrm{(2)}}}\}+\,{\rm{\min }}\{\sqrt{{\chi }_{\hat{B},min}^{s\mathrm{(1)}}},\sqrt{{\chi }_{\hat{B},min}^{s\mathrm{(2)}}}\}\mathrm{.}$$

In this way, we have found a new Entropic Uncertainty Relation. This relation relates the disturbance caused by the measurement of a system’s property to the statistical distinguishability of quantum states. It is important to say that there is not a similar relation in literature, and because of this, we need to associate it with a new statement, namely:

*It is impossible to measure an observable without disturbing simultaneously its probability distribution and the probability distribution of a non-commuting observable*.

Notice that our disturbance-disturbance relation does not apply to the case where the system is in an eigenstate of the measured observable.

In this manner, we have found an uncertainty relation using all the postulates of quantum mechanics, specially the MP. We call it the disturbance-disturbance uncertainty relation (D-D-UR). One of the most important properties of this new D-D-UR is that it is an uncertainty relation measuring distances between probability distributions, see the example given in the second subsection below.

### Some characteristics

Perhaps, as wishful thinking, it is probably expected that if you disturb the probability distribution of $\hat{A}$ just by a little amount, then the resulting disturbance of the probability distribution of $\hat{B}$ will be of a great amount. This expectation comes from the “preparation” uncertainty relation, i.e. Δ*x*Δ*p* ≥ *ћ*//2, where Δ*p* increases by reducing Δ*x*. However, in this case there is a single probability distribution only, and its representation in the configuration space is related by a Fourier transform to its momentum representation. It is a mathematical property that when two functions are related by a Fourier transform when the width of one of them decreases, then the width of the other one increases. Please note that the Measurement Postulate is not used to deduce the “preparation” uncertainty relation.

**Figure 1:**
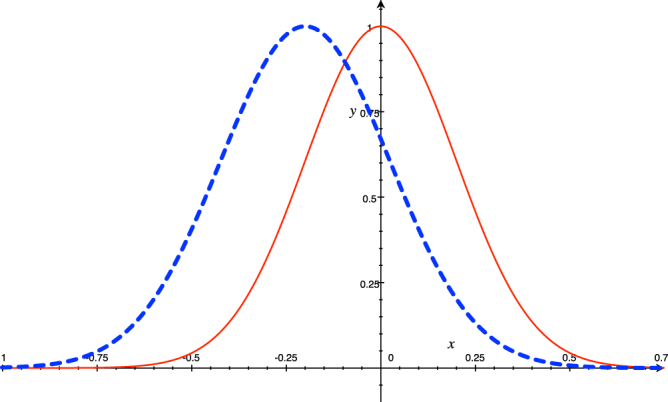
Plot of two wave functions, in blue dash line the initial wave function and in red line the final wave function after a single measurement. Notice that the blue plot is wider than the red one.

In contrast, our disturbance measure is between two probability distributions, one of them before a single measurement and the other one after that single measurement, both of them related by the Measurement Postulate. That is to say, the Fourier transform does not relate them, and it is not possible to expect a priori that they are related in such a way. On the contrary, if the disturbance is on the wave function, what we could expect is: *If we make a little disturbance on the wave function representing the system’s state, then the statistical distribution of its properties also change a little*. This is explained in more detail with the help of the figure 1:

Suppose that the initial state *ϕ*_*i*_(*x*) is given by the blue dash curve in Fig. 1, after the measurement of the observable $\hat{A}$ the wave function collapses towards the wave function in red, *ϕ*_*f*_(*x*), (notice that in this hypothetical case we are trying to consider a situation when the measurement produces a small perturbation on the wave function; of course, you can imagine a better plot with a really minimal perturbation). Then, the statistical distribution of $\hat{A}$ suffers a small change, hence the disturbance is small. But, also, the statistical distribution of a complementary observable changes a little too, because the distance between the initial statistical distribution (in blue), before the measurement, and the final distribution (in red), after the measurement, is small, and this small change in the statistical distribution is for both $\hat{A}$ and $\hat{B}$ . Therefore, it is a naive thinking to expect that, when the statistical distribution changes a little for both observables, whereas the disturbance on $\hat{A}$ decreases, then the disturbance on $\hat{B}$ increases.

It is worth noticing that a “preparation” tradeoff relation is to be expected between two non-commuting observables when the quantum system is in the initial state *ϕ*_*i*_(*x*), i.e. before measurement; or when it is in the final state *ϕ*_*f*_(*x*), i.e. after measurement. In others words, it is expected an usual “preparation” tradeoff relation between two observables when the quantum system is in the state *ϕ*_*i*_(*x*) of Fig. 1, because the state *ϕ*_*i*_(*x*) has a related wave function in the momentum representation *ϕ*_*i*_(*p*) and they are related by *ϕ*_*i*_(*p*) = ∫*e*^−*ixp*/*ћ*^*ϕ*_*i*_(*x*). Then, if you reduce the width of *ϕ*_*i*_(*x*), then the width of *ϕ*_*i*_(*p*) increases. The same applies for *ϕ*_*f*_(*x*) in Fig. 1; also, in this case, there is a tradeoff relation between two complementary observables after the measurement, due to the relation that exist between *ϕ*_*f*_(*x*) and *ϕ*_*f*_(*p*), i.e *ϕ*_*f*_(*p*) = ∫*e*^−*ixp*/*ћ*^*ϕ*_*f*_(*x*); that is, if you reduce the width of *ϕ*_*f*_(*x*), then the width of *ϕ*_*f*_(*p*) increases.

### Example of the disturbance-disturbance uncertainty relation

Suppose that we have a particle of spin $\frac{1}{2}$, we consider that the initial state is $|\varphi \rangle =\frac{1}{\sqrt{2}}(|{x}_{1}\rangle +|{x}_{2}\rangle )$, where |*x*_*i*_〉 is an eigenstate of ${\hat{S}}_{x}$. In this case, we make a projective measurement of the observable ${\hat{S}}_{x}$ on it; consequently, we collapse its state to one of the components of the superposition state. Without loss of generality, suppose that we collapse its state to |*x*_1_〉. By this procedure we have two different statistical distributions of ${\hat{S}}_{z}$, the first |〈*z*_*j*_|*x*_1_〉|^2^ related to the probability of finding the system in a state of spin in *z* after the measurement, and the second |〈*z*_*j*_|*ϕ*〉|^2^ related to the probability distribution before the measurement.

**Figure 2:**
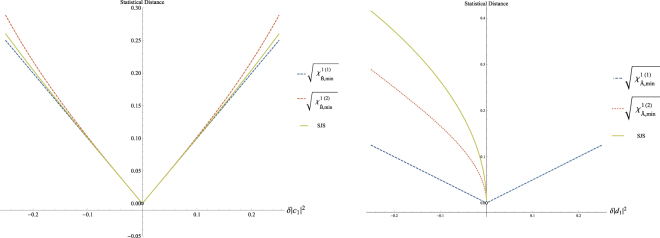
Left: Values of $\sqrt{{\chi }_{\hat{B},min}^{\mathrm{(1)}}}$, $\sqrt{{\chi }_{\hat{B},min}^{\mathrm{(2)}}}$ and SJS when *δ*|*c*_1_|^2^ takes values in the interval [−0.25, 0.25]. Notice that the lines do not reach the origin. Right:Values of $\sqrt{{\chi }_{\hat{A},min}^{\mathrm{(1)}}}$, $\sqrt{{\chi }_{\hat{A},min}^{\mathrm{(2)}}}$ and SJS when *δ*|*d*_1_|^2^ takes values in the interval [−0.25, 0.25]. Notice that the lines do not reach the origin.

Hence, we are going to compare the two probability distributions for the case where the initial state of the system is the nearest state of |*x*_1_〉, that is |*x*_1_〉 + |*δx*_1_〉. The measures that we use are (a) the square root of the Jensen Shanon Entropy and (b) the square root of the *χ* distance. In our example, the distance between probability distributions relies on little variations of the probability distribution due to our asseveration of the nearest state. In this way we can directly apply the equation and obtain the disturbance in the statistic of the observable ${\hat{S}}_{z}$

23 $${D}_{({\hat{S}}_{z})}\ge \,{\rm{\min }}\{\sqrt{{\chi }_{\hat{B},min}^{s\mathrm{(1)}}({S}_{z})},\sqrt{{\chi }_{\hat{B},min}^{s\mathrm{(2)}}({S}_{z})}\}$$

where the Jensen-Shanon entropy is given by:

24 $${D}_{({\hat{S}}_{z})}=\sqrt{\sum _{j=1}^{N}(|\langle {z}_{j}|{x}_{1}\rangle {|}^{2}\,\mathrm{ln}\,\frac{2|\langle {z}_{j}|{x}_{1}\rangle {|}^{2}}{|\langle {z}_{j}|{x}_{1}\rangle {|}^{2}+|\langle {z}_{j}|\varphi \rangle {|}^{2}}+|\langle {z}_{j}|\varphi \rangle {|}^{2}\,\mathrm{ln}\,\frac{2|\langle {z}_{j}|\varphi \rangle {|}^{2}}{|\langle {z}_{j}|\varphi \rangle {|}^{2}+|\langle {z}_{j}|{x}_{1}\rangle {|}^{2}})};$$

where $\sqrt{{\chi }_{\hat{B},min}^{s\mathrm{(1)}}({S}_{z})}=\delta |{c}_{1}{|}^{2}$, and $\sqrt{{\chi }_{\hat{B},min}^{s\mathrm{(2)}}({S}_{z})}=\frac{1}{2}\sqrt{\frac{{(\delta {|{c}_{1}|}^{2})}^{2}}{\frac{1}{4}-{(\delta {|{c}_{1}|}^{2})}^{2}}}$. We have written $|{x}_{1}\rangle =\frac{1}{\sqrt{2}}(|{z}_{1}\rangle +|{z}_{2}\rangle )$, and use the condition *δ*|*c*_1_|^2^ = *−δ*|*c*_2_|^2^ due to the requirement of normalization. Remember that *δ*|*c*_*j*_| = 〈*z*_*j*_|*δx*_1_〉. At the left of Fig. (2), we can see the square root of *χ* and between these the square root of the Jensen-Shannon entropy (SJS).

On the other hand, we are going to compare the two probability distributions of *S*_*x*_; in our example, these distributions are |〈*x*_1_|*x*_1_〉|^2^ = 1, the distribution after the measurement; and |〈*x*_*j*_|*ϕ*〉|^2^, the distribution before the measurement. It is crucial to think deeply about this part of the example because in this case we are comparing a delta distribution with a distribution close to the delta distribution. We have the normalization condition that allows positive and negative values of the little fluctuations, i.e. (*δ*|*d*_*j*_|^2^), and compensates to leave the probability of the distribution before the measurement intact.

After calculations we obtain: $\sqrt{{\chi }_{\hat{A},min}^{s\mathrm{(1)}}({S}_{x})}=\frac{1}{2}\delta |{d}_{1}{|}^{2}$, and $\sqrt{{\chi }_{\hat{A},min}^{s\mathrm{(2)}}({S}_{x})}=\frac{1}{2}\sqrt{(1+\delta {|{d}_{1}|}^{2}){(1-\frac{1}{1+\delta {|{d}_{1}|}^{2}})}^{2}-\delta |{d}_{1}{|}^{2}}$, under similar conditions, where *δ*|*d*_*j*_| = 〈*x*_*j*_|*δx*_1_〉.

At the right of Fig. (2) we see the different *χ* distances and the square root of the Jensen-Shannon entropy (SJS). We note, from the equations of these quantities, that SJS and the *χ* distance are not defined for positive *δ*|*d*_*j*_|^2^, i.e. they become imaginary, in this way these two distances are not suitable since they do not satisfy the normalization condition.

In Fig. (2) we gave different values for *χ* and SJS distances to show its behaviour, however, it is not because they get different values. In fact, these quantities have a single value given by the distance between the nearest distinguishable states after *n* trails. By changing their values we can see how they behave when the capacity to statistically distinguish two states becomes optimal.

## Discussion

The uncertainty relation that we are presenting differs from the ones already known because it quantifies the disturbance caused in the statistical distributions, whereas others focus on the relations between noise and disturbance in the measurements^22,23,32,40,41^. The usual uncertainty relation by Kennard and Robertson^44^ is about the statistics as a result of the preparation of quantum states, i.e. it limits the prior knowledge of the statistics of the observables and its predictability^3^, whereas the D-D-UR includes in its derivation the process of measurement by taking into account the MP.

The D-D-UR also differs from the kind of uncertainties related to complementarity^3^ because of the impossibility to arrange an experiment which could measure the value of complementary observables, certainly we could generalise our results to include this kind of uncertainty also.

In a recent work, Shitara *et al*.^29^ discussed an inequality given by Barchielli and Lupieri^30^, this inequality interpreted as an information-disturbance relation. Then, by choosing two near states as the argument of the relative entropy, the main results of Shiatara *et al*. coincide^29^ with that obtained by Barchielli and Lupieri^30^. The generalisation in these works consist in translating the inequality obeyed by the relative entropy ${S}^{C}(p||q)\le {S}^{Q}(\hat{\rho }||\hat{\sigma })-{\sum }_{i}{p}_{i}{S}^{Q}({\hat{\rho }}_{i}||{\hat{\sigma }}_{i})$ to an inequality obeyed by a divergence ${D}^{C}(p||q)\le {D}^{Q}(\hat{\rho }||\hat{\sigma })-{\sum }_{i}{p}_{i}{D}^{Q}({\hat{\rho }}_{i}||{\hat{\sigma }}_{i})$. In this case, it is worth mentioning that the relative entropy is not symmetric, which mean that it is not a proper distance between two probability distributions, it seems that this also occur with the classical divergence. In our work, we restrict ourselves to the square root of the Jensen-Shannon divergence which represents a truly metric, since it is symmetric and obeys the triangle inequality.

As a conclusion, to the best of our knowledge, the D-D-UR was not previously proposed and it refers to limitations on knowledge and predictability of the value of an observable $\hat{B}$ before and after the measurement of another observable $\hat{A}$ and vice versa.

## Methods

From the equations of Braunstein and Caves^72^, we can write the nearest state to |*ψ*〉, when |*ψ*〉 tends to |*a*_*s*_〉, as:

25 $$|{\psi }_{N}\rangle =|{a}_{s}\rangle +|\delta {a}_{s}\rangle =\sum _{i=1}^{n}\sqrt{{p}_{i}+\delta {p}_{i}}\exp ({\varphi }_{j}+\delta {\varphi }_{j})|{v}_{i}\rangle ,$$

where |*v*_*i*_〉 is a basis vector. Writing this equation with the above notation we have:

26 $$|{\psi }_{N}\rangle =\sum _{i=1}^{n}\sqrt{|{c}_{i}{|}^{2}+\delta |{c}_{i}{|}^{2}}\exp ({\varphi }_{j}+\delta {\varphi }_{j})|{v}_{i}\rangle ,$$

Now, the normalization condition implies that $\langle {\psi }_{N}|{\psi }_{N}\rangle =1={\sum }_{i=1}^{n}(|{c}_{i}{|}^{2}+\delta |{c}_{i}{|}^{2})$. As, ${\sum }_{i\mathrm{=1}}^{n}|{c}_{i}{|}^{2}=1$, therefore

27 $$\sum _{i=1}^{n}\delta |{c}_{i}{|}^{2}=\sum _{i=1}^{n}\delta {p}_{i}=0.$$

We can see from Eq. (27) that variations in the probability distribution are compensated due to the normalization of the probability, ${\sum }_{i=1}^{n}|{c}_{i}{|}^{2}={\sum }_{i=1}^{n}|\langle {v}_{i}|{a}_{s}\rangle {|}^{2}=1$. Note that the variation of the probability distribution, *δp*_*i*_, can be positive or negative.
